# Knowledge Spillover and Innovation in Healthcare: A Comparative Study of Social Media Interaction Between Medical Technology Companies and Healthcare Professionals in Austria

**DOI:** 10.3390/jmahp14010001

**Published:** 2025-12-22

**Authors:** Mariella Zilahi-Lugbauer, Harald Stummer

**Affiliations:** 1 Department of Economic and Social Sciences, University Seeburg Castle, Seeburgstr. 8, 5201 Seekirchen am Wallersee, Austria; 2 Institute for Management and Economics in Healthcare, UMIT Tyrol, 6060 Hall in Tyrol, Austria; harald.stummer@umit-tirol.at; 3 Institute for Health Management, IMC Krems, 3500 Krems an der Donau, Austria

**Keywords:** knowledge spillover, information-processing capability, involvement, innovation performance, social media, strong and dense ties

## Abstract

***Background***: Interactions between healthcare professionals and medical technology companies on social media are central to understanding how variations in knowledge spillover and innovation performance vary. ***Aim***: This study investigates how social media facilitates knowledge exchange between these two stakeholder groups in Austria, drawing on a cross-sectional online survey of 97 participants (45 healthcare professionals and 52 medical technology company representatives). Data were analyzed using Confirmatory Factor Analysis (CFA), Exploratory Structural Equation Modeling (ESEM), and independent-samples *t*-tests. The comparative approach enabled the identification of structural differences across stakeholder groups and regions within Austria. ***Methodology***: The study adopts a comparative analysis framework to explore geographic differences within Austria and to examine how social media interaction strengthens dense and strong network ties between healthcare professionals and medical technology companies, thereby enhancing information-processing capabilities. ***Results***: The findings underscore the pivotal role of social media in bridging geographic boundaries, fostering dense and strong network ties, and enhancing information-processing capacities. ***Conclusions***: This study advances the understanding of how digital interaction mechanisms shape knowledge exchange and innovation performance in healthcare outcomes. ***Practical Implications***: The findings suggest that social media promotes stronger professional relationships and deeper customer engagement. The results may assist policymakers and industry leaders aiming to design effective digital strategies for innovation and improved healthcare outcomes.

## 1. Introduction

### 1.1. Background

Innovation in healthcare depends increasingly on effective knowledge exchange between different actors such as healthcare professionals, medical technology companies, and policy stakeholders [1].

Digital platforms, and especially social media, now represent important vehicles for this exchange because they allow professionals to share experiences, clinical insights, and technological developments beyond institutional boundaries [2].

In Austria, the digital transformation of professional communication has accelerated but remains under examined from a knowledge-management perspective [3].

### 1.2. Knowledge Spillover in Healthcare

Knowledge spillover describes the process through which knowledge generated by one organization or individual becomes accessible and beneficial to others without direct compensation [4].

In healthcare, such spillovers can occur through conferences, joint projects, publications, and, with increasing frequency, through social media interaction [5].

This study builds on the knowledge-based view (KBV) [6], which conceptualizes knowledge as the key strategic resource driving innovation and organizational performance.

According to KBV, social media platforms may act as boundary-spanning mechanisms that facilitate the transfer of tacit and explicit knowledge between healthcare professionals and industry representatives [7].

### 1.3. Social Media and Innovation

The role of social media in healthcare innovation has been discussed as a double-edged phenomenon: while it enables rapid dissemination of clinical knowledge, it also poses challenges related to data quality, privacy, and credibility [8].

Previous research has shown that digital interaction supports open innovation [9], collaborative learning [10], and knowledge dissemination [11].

However, few empirical studies have directly compared healthcare professionals and medical technology companies in Central Europe [12].

### 1.4. Research Gaps and Objectives

Although several studies have examined social media use in healthcare communication [13,14,15], few have analyzed how such interactions contribute to knowledge spillover and innovation performance. Moreover, the comparative perspective between healthcare professionals and medical technology companies remains insufficiently addressed.

Therefore, this study investigates how differing social media usage patterns affect the acquisition, dissemination, and utilization of knowledge among both groups [16].

The primary objective is to determine whether these interactions lead to measurable differences in perceived innovation performance [17].

By comparing two central stakeholder groups within Austria’s healthcare sector, this study clarifies how digital communication dynamics influence innovation capacity. Building on this conceptual foundation, the following section develops the theoretical framework underpinning the study’s hypotheses.

## 2. Theory

The knowledge-based view (KBV) conceptualizes knowledge as the primary strategic resource driving organizational innovation. From this perspective, a competitive advantage arises from companies’ ability to acquire, integrate, and apply knowledge effectively across boundaries. Social media enables such cross-boundary knowledge flows by facilitating real-time interaction between heterogeneous actors, thereby enhancing absorptive capacity [7].

Strong and dense ties, central constructs of Social Network Theory, facilitate rich, frequent, and high-quality exchanges of knowledge and experience [8]. Within the KBV framework, these ties are fundamental for effective knowledge transfer, as they allow us to access and leverage deep, tacit knowledge that cannot be obtained through weak ties.

Information-processing capabilities, which consist of acquisition, dissemination, and use of customer information, are directly aligned with the KBV, as they involve processes essential for managing knowledge for both subject groups [3]. The acquisition of customer information involves gathering valuable data that can be transformed into actionable knowledge, while dissemination refers to its internal and external sharing among stakeholders, and the use of customer information describes how effectively such knowledge is employed to drive innovation. Social media platforms function as powerful enablers of interactive knowledge exchange [4]. They enhance information-processing capabilities by fostering direct feedback loops, dialogue, and engagement, thereby promoting the acquisition, dissemination, and application of knowledge. Such interaction offers the opportunity to tailor innovation and improve products in alignment with customer needs as a pre-condition for knowledge spillover.

The relatively slow diffusion of new technologies and practices within the healthcare sector, combined with limited understanding of how social networks support professional knowledge-sharing [6], motivated this research. Moreover, the low rate of social media interactions between the two groups [9], despite their simplicity and cost-effectiveness, highlights the untapped potential for knowledge-sharing and networking. Few studies have examined opinions on the use of social media for knowledge-sharing in health and medical technology [6]. The underlying reasons for the slow adoption and diffusion of innovation remain insufficiently examined, indicating a significant gap in the literature.

### 2.1. Knowledge Spillover Through Social Media

Knowledge spillover is defined as the exchange of unintentional ideas between individuals [10], which can occur through both formal and informal cooperation with customers, both of which are valuable. Knowledge flow increases absorptive capacity [11] and results in knowledge spillover [12]. In healthcare, knowledge spillover through social media involves the transfer of knowledge between the two groups that catalyzes innovation in treatment methods, diagnostic and surgical techniques, and new product development [13].

Mohnen [14] evaluated the effects of healthcare professional’s knowledge spillover and determined that knowledge sharing and networking are essential for them to successfully fulfill their professional work. Integrating healthcare professionals into the innovation process is essential for medical technology companies to meet market demands and address upcoming customer needs. Social media serves as a communication channel for real-time knowledge exchange [4], facilitating knowledge spillover [15]. Many studies have called for future studies to explore the utilization of social networks as a medium for knowledge exchange in the medical field [6].

Recent studies confirm that social media is a knowledge-sharing tool, revealing different ways of sharing; this makes it challenging to define a theoretical framework, suggesting further research is needed to develop one [16]. This highlights a broader research gap in innovation management theory, particularly concerning how network-based knowledge sharing and cooperation contribute to product and process innovation [17]. Building on these insights, this study investigates how social media-enabled knowledge spillover affects innovation performance in healthcare and whether these effects differ between healthcare professionals and medical technology companies [18].

On this basis, we hypothesize the following:

**H1.1:**  *Healthcare professionals and medical technology companies differ in terms of knowledge spillover effect.*

### 2.2. Innovation Performance Through Social Media

Innovation performance through social media interaction refers to the extent to which engagement on digital platforms contributes to the generation, development, and implementation of new ideas, technologies, and processes within healthcare organizations. Such interaction enhances knowledge flow across institutional boundaries, enabling the timely dissemination and integration of novel insights into clinical and technological practice [19].

In healthcare, innovation performance is facilitated by social media interactions. Knowledge flow, including knowledge sharing, transfer, conversion, and integration, refers to the application and development of knowledge over time. Knowledge inflow through involvement manifests in three forms: as an information source, a co-developer source, and an innovation source.

Although prior research has examined the relationship between innovation performance and knowledge spillover, the interplay between spillover mechanisms, cooperation intensity, and innovation outcomes remains insufficiently understood, particularly within the healthcare sector. Given this conceptual gap, this study investigates how social media-enabled knowledge exchange contributes to innovation performance and how cooperation dynamics mediate this relationship.

Therefore, we hypothesize the following:

**H1.2:**  *Healthcare professionals and medical technology companies differ in terms of innovation performance.*

### 2.3. Information-Processing Capabilities Through Social Media

The idea of information-processing capability, discussed by [20], is vital for understanding how companies can sustain an advantage in the market. Dynamic capabilities refer to companies’ capacities to absorb and adjust both internal and external knowledge in dynamic settings [20]. The two groups differ in knowledge absorption and interaction patterns, influencing knowledge spillover [21].

The term information-processing capabilities refers to an organization’s ability to acquire, disseminate, and use information to create value. This allows companies to make on-the-fly adjustments based on customer feedback [22].

Active engagement through social media platforms significantly enhances the information-processing capacity of both groups. Healthcare professionals, in particular, benefit from these interactions by accessing professional expertise, sharing clinical experiences, and participating in co-development discussions that enhance their own learning and innovation potential [23]. Such reciprocal exchanges strengthen the knowledge infrastructure that supports innovation performance and continuous improvement within healthcare ecosystems.

Cumulative evidence underscores the critical role of information-processing capabilities and the enabling influence of social media in fostering collaborative innovation and product development between healthcare professionals and medical technology companies. Thus, we hypothesize the following:

**H2.1:**  *Healthcare professionals and medical technology companies differ in terms of information processing.*

### 2.4. Involvement Through Social Media

Engaging customers directly in the innovation process through social media platforms enables organizations to access substantial inflows of external knowledge and real-time feedback, thereby enhancing new product development and positively influencing innovation performance [3,12,19,24]. Recent research highlights the significance of new external knowledge inflow [19] and the effectiveness of involvement via social media [4,24] due to the additional knowledge resources it provides, thereby benefiting companies significantly.

Research suggests that knowledge among healthcare professionals often flows informally and is characterized by exchanges of information between colleagues. Healthcare professionals can generally be categorized within their sub-disciplines, influenced primarily by their attitudes and individual experiences [25].

Halbusi et al. (2022) [26] emphasized the importance of identifying factors that shape the effectiveness of customer interaction in innovation processes and called for further investigation into the determinants of meaningful customer involvement in product development [3]. Moreover, a deeper understanding of what constitutes customer participation in innovation along with the challenges of knowledge synthesis, such as user motivation, capacity to contribute, and the absorptive ability of companies remains underdeveloped in the current literature [19]. Thus, we establish the following hypothesis:

**H2.2:**  *Healthcare professionals and medical technology companies differ in terms of involvement on social media.*

### 2.5. Dense and Strong Ties in Social Media

Networks consist of strong and weak ties [27] each contributing uniquely to the formation of relationships and the facilitation of knowledge flow and spillover [12]. Strong ties involve continuous, frequent interactions characterized by mutual trust, reciprocity, and long-term engagement, thereby fostering a stable environment for the exchange of complex or tacit knowledge. In contrast, weak ties according to Granovetter’s seminal theory serve as bridges that connect otherwise unlinked social systems, enabling access to novel information and external perspectives [27]. Dense ties are representing closely knit clusters within networks and were originally conceptualized by [8], and later redefined by [12]. These dense ties play a key role in building trust and cooperation, which enhances the potential for effective knowledge exchange and collaboration. Dense ties affect the relationships between innovators and the innovation process [8]. Researchers state that strong-tied networks are favored over new contacts, as strong relationships and better common understanding between stakeholders, leading to weighty knowledge transfer, take them into consideration. However, only a few studies have evaluated the two groups’ opinions about using social media for knowledge exchange in the medical field [6].

A deeper understanding of the various relationships within the innovation process could be useful. It is important to determine whether weak or strong ties are more pivotal for knowledge spillover [12]. Given the dynamic and interactive nature of social media, it is plausible that the two groups experience distinct effects from network ties: strong ties may promote trust and collaboration, whereas dense ties could accelerate information flow and learning. This reasoning underpins our assumption that the strength and density of network ties may vary across professional contexts, influencing the effectiveness of social media-enabled knowledge exchange. Thus, we establish the following hypothesis:

**H3:**  *Healthcare professionals and medical technology companies differ in terms of the effect of strong and dense ties.*

## 3. Method

### 3.1. Study Design

This study follows a quantitative approach, utilizing a cross-sectional design to investigate the behaviors and attitudes of two specific groups in Austria. A cross-sectional design was deliberately chosen because it allows for the efficient examination of existing relationships between constructs at a single point in time, without the confounding effects of temporal variation. This approach is appropriate for exploratory and comparative research such as the present study, which aims to identify structural differences between healthcare professionals and medical technology companies. In addition, the cross-sectional survey enabled access to a geographically dispersed population within a limited time frame and resource setting, which is a common constraint in healthcare field research. Data were gathered using SoSi Survey between 13 April and 13 May 2024. The online survey design enabled effective access to Austria’s geographically dispersed population and provided valuable insights into behavioral patterns within a distinct cultural and socio-economic context, making Austria an appropriate and compelling case study. A non-probability convenience sampling approach was used, which may introduce selection bias and restrict generalizability.

### 3.2. Questionnaire

All constructs in this study were measured using adapted, previously validated scales to ensure reliability and comparability across domains.

Knowledge spillover effects were operationalized using six items adapted from Wang and Jiang and measured with a seven-point Likert scale [3,13].

Information-processing capabilities were examined using an adapted version of the scale described in [3]. This construct includes the acquisition, dissemination, and the use of customer information and was assessed using a twelve-item instrument with a seven-point Likert scale. The foundational scale was originally developed by Veldhuizen, Hultink and Griffin.

A three-item adapted scale with a five-point Likert scale for evaluating innovation performance was used to measure the differentiated assessment of innovation performance [12].

Participant involvement was measured using a four-item measure adapted from the scale created by [19], including the modifications made by [3]. This seven-point Likert scale evaluated the level of social media participant involvement.

This research explored the characteristics of social networks, focusing on dense and strong ties within professional networks. A four-item adapted scale using a five-point Likert scale was engaged to measure both types of ties [12].

For the item “geographic region,” the study targeted the nine federal states of Austria, surveying the locations of company headquarters. A distinction was postulated between rural and urban regions [23]. Rural areas were defined as those with populations below 10,000 inhabitants. This geographical categorization enabled the analysis of how regional context influences knowledge-exchange dynamics.

### 3.3. Questionnaire Development and Pretests

The online survey was administered in German to ensure accessibility for participants in Austria. To guarantee linguistic accuracy and conceptual equivalence of the translated instrument, Brislin’s back-translation procedure was applied [28]. First, a bilingual professional translated the English survey into German, after which an independent translator performed a reverse translation into English. Discrepancies between the original and back-translated versions were reviewed and resolved through consensus to preserve semantic integrity.

Pretesting was conducted with three participants from each target group (healthcare professionals and medical technology company representatives) to assess construct clarity and response comprehension. Based on their feedback, minor linguistic and structural adjustments were implemented.

Subsequently, a pilot test involving 30 participants from each group was carried out to further evaluate the survey’s reliability and validity, taking into account demographic diversity. Completion rates and open-ended participant comments were analyzed to assess response quality and identify potential issues with item interpretation, contributing to the overall improvement of the survey’s content validity.

### 3.4. Sampling Technique and Data Collection

Surveys invitations were distributed to 1063 healthcare professionals and 554 representatives of medical technology companies, using publicly available contact information from the Herold telephone directory in Austria. This two-pronged sampling approach enabled the inclusion of both key stakeholder groups, thereby ensuring comprehensive coverage of the Austrian healthcare innovation ecosystem.

Data analysis was performed using IBM SPSS Statistics Version 28, JASP Version 0.18.3, Mplus Version 8.11, and G*Power Version 3.1.9.6. Descriptive statistics were calculated to summarize central tendency and dispersion parameters. The Shapiro–Wilk test, supported by visual inspections of histograms, Q–Q plots, and boxplots, was employed to assess the normality of distributions.

After data collection, 54 incomplete responses identified during preliminary quality checks were excluded. Consequently, the initial gross sample of 233 responses was refined to a final analytical sample comprising 97 fully completed and valid questionnaires. This final sample size was sufficient for the planned group comparisons and for the calculation of factor-analytic models.

## 4. Analyses

To ensure methodological transparency, this section outlines the sequence of statistical procedures used to evaluate construct validity, reliability, and group differences. When the assessment of normal distribution check is ambiguous, the findings of the parametric and, if applicable, non-parametric tests are presented in this section. This parallel reporting increases the robustness of the conclusions. Moreover, the parametric *t*-test, Levine’s test, and, where appropriate, the non-parametric Mann–Whitney U-test were used. A two-sided hypothesis test was conducted in each case.

The correlations were calculated using either Pearson’s r or Spearman’s rho, depending on the prerequisite test. Binomial and chi-square tests were also performed to examine relationships among categorical variables. In detail, the following analyses were carried out: Confirmatory Factor Analysis (CFA), CFA with a Maximum Likelihood Estimator and bootstrapping to test factorial validity; Exploratory Structural Equation Modeling (ESEM) to examine dimensionality and cross-loadings; Latent Moderated Structural Equations (LMSs) to test interaction effects; and reliability was mapped using Cronbach’s alpha.

By combining CFA, ESEM, and LMSs, this study integrates confirmatory and exploratory perspectives and thereby strengthens the validity of the measurement model.

## 5. Results

### 5.1. Descriptive Analysis of the Sample

In the final sample, 97 participants were included, consisting of 45 healthcare professionals and 52 representatives of medical technology companies. This distribution ensured that both stakeholder perspectives were adequately represented.

The age distribution of the study participants skewed toward older age groups.

Males dominated the sample. Out of 97 participants, 26 (26.80%) identified themselves as female and 66 (68.04%) were male. This demographic pattern reflects the current structure of the Austrian medical and medical technology sectors and should be considered when interpreting the results.

### 5.2. Construct Factorial Validation, Reliability, and Comparisons

The interpretation of the values was derived based on the limit values. The use of the measurement instrument for knowledge spillover shows the good model performance in this study. The model performed better for all parameters than in the original study [13]. Table 1 shows an overview of the characteristics. All major fit indices (CFI, TLI, RMSEA, and SRMR) fell within acceptable or good ranges, confirming the adequacy of the German version of the scales.

The model established in [12] for strong and dense ties showed a poor model fit in terms of factorial validity in the original version, but the present study achieved clearly improved fit indices, indicating that the adapted items are suitable for the Austrian context. This is reflected in the good to very good Cronbach’s alpha values derived in the analysis. Table 2 provides an overview of the characteristics.

The customer involvement constructs were originally planned to be calculated using Latent Moderated Structural Equations (LMSs). However, only one scale from Cui and Wu was included in the final study [19]. Given the limitations of a three-item Confirmatory Factor Analysis, an Exploratory Factor Analysis was conducted instead. The principal component method with varimax rotation and an eigenvalue criterion greater than 1 and the scree plot criterion were applied. The MSA (Kaiser–Meyer–Olkin test) measure of sampling adequacy was 0.760, with item-level values of 0.846, 0.739, and 0.713, indicating good factorability. Bartlett’s test of sphericity confirmed sufficient intercorrelation among items. All loadings exceeded 0.80, supporting the assumption of a single latent dimension. The first factor accounted for 85.6% of the total variance, and the internal consistency was excellent (Cronbach’s α = 0.946).

ESEM results for the acquisition, dissemination and use of information (Table 3) demonstrated a strong model fit. The χ^2^/df of 1.44 compared to 2.01 in [3]. RMSEA and SRMR values indicated a good fit and GFI values exceeded 0.90, confirming the three-dimensional structure of information-processing capability in this sample.

The GFI reported by [3] met the recommended threshold, although corresponding values were not available for the present study (2024). The current analysis demonstrated satisfactory reliability across all constructs. Average variance extracted (AVE) values exceeded 0.50 for the dissemination and use of information dimensions, while the acquisition of information dimension was marginally below this threshold, yet still within an acceptable range.

### 5.3. Hypothesis Testing

To evaluate the six hypotheses (H1.1, H1.2, H2.1, H2.2, H3.), group comparisons between healthcare professionals and medical technology company representatives were conducted using *t*-tests and, where appropriate, Mann–Whitney U tests.

#### 5.3.1. Hypothesis 1.1: Knowledge Spillover Effect

Based on the results of the prerequisite tests, both a robust Welch test and the Mann–Whitney U test showed no significant difference between the two groups (t(93.054) = −0.193, *p* = 0.847; U = 1174.000, *p* = 0.980).

This means that, despite their different roles in the healthcare ecosystem, both groups perceive knowledge spillover via social media at a comparable level.

#### 5.3.2. Hypothesis 1.2: Innovation Performance

The Mann–Whitney U test likewise revealed no significant difference (U = 1166.500, *p* = 0.982).

Thus, innovation performance driven by social media interaction appears to be similarly perceived by both groups.

#### 5.3.3. Hypothesis 2.1: Acquisition of Customer Information, Dissemination of Customer Information, and Use of Customer Information

Student’s *t*-test showed a significant difference only for the acquisition of customer information: t(95) = −2.368, *p* = 0.020, d = −0.482; Mann–Whitney U = 859.500, *p* = 0.025, r = −0.265.

Medical technology company representatives reported higher levels of information acquisition than healthcare professionals.

No significant differences were found for dissemination (t(95) = −0.185, *p* = 0.853) or use of customer information (t(95) = −1.763, *p* = 0.081).

This suggests that once information is acquired, both groups process and apply it in a similar manner.

#### 5.3.4. Hypothesis 2.2: Customer Involvement (CI)

The Mann–Whitney U test revealed a significant group difference, with a small effect size, in customer involvement scores between the two groups (U = 815.000, *p* = 0.010, r = −0.303). This indicates that medical technology companies maintain higher levels of social media-based involvement than healthcare professionals.

#### 5.3.5. Hypothesis 3: Strong and Dense Ties

A Student’s *t*-test showed a significant difference in strong ties (t(95) = −2.767, *p* = 0.007, d = −0.563), indicating a medium effect size. The Mann–Whitney U test corroborated this result (U = 823.500, *p* = 0.012, r = −0.296). These findings suggest that medical technology company representatives maintain stronger and more cohesive professional connections through social media than healthcare professionals.

A Student’s *t*-test also revealed a significant difference in dense ties (t(95) = −2.234, *p* = 0.028, d = −0.455), representing a medium effect size. This indicates that company representatives exhibit denser network structures compared with healthcare professionals, reflecting more frequent and interconnected social media-based exchanges within their professional communities.

## 6. Discussion

This section interprets the empirical findings in light of the theoretical framework developed earlier, linking them explicitly to the knowledge-based view (KBV) and Social Network Theory.

### 6.1. Validity and Reliability

The assessment of factorial validity and reliability consistently demonstrated comparable or superior model quality relative to the original validation studies. This confirms that the German-language adaptations of the English measurement instruments used in this study were psychometrically sound and appropriate for application in German-speaking countries. Thus, the measurement approach appears suitable for future healthcare innovation studies in Austria and comparable contexts.

### 6.2. Theoretical Implications

The results of this study indicate no significant difference in the overall knowledge spillover effect between healthcare professionals and medical technology company representatives. This finding aligns with the knowledge-based view (KBV), which emphasizes that effective knowledge sharing is essential for leveraging organizational capabilities and achieving competitive advantage [7].

When digital channels reduce access barriers, both professional groups can reach a comparable level of knowledge inflow.

The outcomes reveal no discrepancy in innovation performance between the two groups, hinting at comparable results derived from utilizing social media for innovation by both parties involved. The discovery is consistent with studies by [19,24], which emphasize the effectiveness of social media in fostering innovation across different professional settings. The absence of significant differences for H1.1 and H1.2 may reflect similar social media usage patterns and comparable information-exchange behaviors of both groups, as well as the relatively small and homogeneous Austrian sample.

By contrast, there is a significant difference in the acquisition of customer information, with medical technology company representatives recording higher levels.

This indicates that medical technology company representatives are more practiced at gathering valuable customer insights through social media.

This supports the knowledge-based view [7] and Social Network Theory [27], both of which state that organizations with more developed absorptive capacities benefit more from identical external knowledge environments.

The higher customer involvement scores among medical technology company representatives further highlight their stronger focus on engaging customers through social media. Such engagement is essential for integrating customer feedback into the innovation process [4]. The significant difference underscores a potential competitive advantage for medical technology companies. In contrast, healthcare professionals appear to play a more limited role in social media-based knowledge sharing [29].

The significant differences in both dense and strong ties, with company representatives again scoring higher, indicate that these individuals maintain more numerous and robust interactions within their professional networks. From the perspective of network theory, strong ties are essential for building trust and enabling the transfer of complex, tacit knowledge, whereas dense ties foster frequent and efficient communication. These findings are consistent with prior research [12,27], and highlight the importance of social network structures in enabling knowledge transfer and innovation.

Combining insights from knowledge spillover and network theory provides an integrated understanding of how social media can be leveraged to improve innovation. Knowledge spillover highlights the position of accessible communication channels, while network theory underscores the importance of relationship quality and structure in facilitating knowledge flow. This integrative perspective strengthens the conceptual link between digital collaboration and innovation outcomes.

Overall, the findings reinforce theoretical assumptions on knowledge spillover and network theory, underscoring social media’s central role in knowledge transfer and the value of strong network structures in realizing these benefits. Nevertheless, the Austrian context may limit the generalizability of these settings with different digital infrastructures or healthcare systems.

### 6.3. Practical Implications

The findings of this study provide several practical avenues for enhancing knowledge sharing between healthcare professionals and medical technology companies through social media.

First, strengthening and densifying professional network ties is essential. For healthcare professionals, this involves actively engaging in digital professional groups, online forums, and academic discussions where medical technology innovations and practices are shared.

Medical technology companies should leverage their current networks to conserve strong and dense ties with healthcare professionals. Employing targeted social media campaigns to solicit feedback and proposals can improve the knowledge acquisition process.

Third, both stakeholders should focus on effective knowledge acquisition and dissemination. Healthcare professionals can stay updated with the state-of-the-art medical technology advancements by following leading companies, joining relevant online communities, and contributing actively to professional updates, publications, and newsletters.

By adopting these practices, both groups can foster a more collaborative, innovative, and knowledge-driven healthcare environment.

### 6.4. Limitations and Future Perspectives

This research has limitations. The study’s small sample size may constrain the generalizability. The unequal representation of healthcare professionals (46%) and medical technology company representatives (54%) and the modest sample size (n = 97) limit the statistical power and generalizability. Because of the cross-sectional design, causal relationships and temporal dynamics between knowledge spillover and innovation performance cannot be inferred. Longitudinal designs could clarify these relationships and show how digital knowledge networks develop over time. Upcoming research should include larger, more varied samples to authorize these results across diverse settings and cultures, evaluating consequences on social media-driven knowledge spillover and innovation. Future research should employ larger, more diverse samples and mixed-method designs to explore contextual moderators, such as digital literacy, institutional support, and data governance that shape social media-driven innovation.

As data were self-reported, the results may be subject to common-method variance or social desirability bias, particularly regarding innovation-related behaviors.

Organizational beliefs and specific attitudes toward the effects of social media on knowledge sharing deserve further investigation. Supportive organizational principles maximize social media’s potential for knowledge transfer and innovation.

A deeper understanding of these influences, combined with paying attention to the perspectives and behaviors of individual stakeholders, can inform the design of targeted strategies that enhance the effectiveness of social media in promoting organizational learning and knowledge exchange. This is especially pertinent in healthcare, where timely and efficient knowledge sharing directly drives technological development, innovation, and sustained competitive advantage.

## 7. Conclusions

This study provides empirical evidence of how social media interaction facilitates knowledge spillover and innovation performance using Austria’s healthcare ecosystems as an example. By comparing healthcare professionals and medical technology companies, it demonstrates that digital communication platforms are central enablers of cross-sectoral knowledge exchange. Although both groups exhibit similar levels of knowledge spillover and network density, they differ significantly in their information-processing capabilities and degrees of involvement.

Healthcare professionals predominantly utilize social media to acquire and exchange clinical knowledge, whereas medical technology companies employ these platforms strategically to disseminate information and foster collaborative activities.

These results advance the knowledge-based view (KBV) and Social Network Theory by illustrating that social media platforms operate as boundary-spanning mechanisms, enabling the transformation of individual learning processes into collective innovation outcomes.

Furthermore, the findings emphasize that the strength and quality of network ties, rather than their mere quantity, help to decide the effectiveness of digital knowledge exchange. From a policy perspective, encouraging structured and secure professional networking environments could accelerate innovation diffusion and strengthen cooperation between healthcare and industry stakeholders. Managers could invest in digital literacy programs and knowledge-management frameworks to enhance the absorptive capacity of organizations and professionals alike.

Future research should employ longitudinal or mixed-method designs to uncover causal dynamics and contextual moderators influencing digital knowledge transfer.

All in all, digital knowledge transfer should be seen not merely a communication medium but as transformative infrastructure for knowledge integration, learning, and innovation in healthcare.

## Figures and Tables

**Table 1:** Comparison of the validity and reliability of knowledge spillover.

Knowledge Spillover (CFA)	χ^2^	χ^2^/df	RMSEA	NFI	CFI	IFI	TLI	Cronbach Alpha
Models								
Wang and Jiang 2020 [13] ^1^	χ^2^ = 179.14 *p* = 0.01		RMSEA = 0.07	NFI = 0.92	CFI = 0.93	IFI = 0.91		0.752
Present study 2024 ^2^	χ^2^ = 8.157 DF = 6 *p* = 0.227	χ^2^/df = 1.36	RMSEA = 0.061 CI-90 [0.000; 0.154] *p* = 0.365	NFI = 0.98	CFI = 0.99	IFI = 0.99	TLI = 0.989	0.933
Comparison of knowledge spillover			deltaRMSEA = 0.009			deltaCFI = −0.06		
Factor loading—AVE								
Wang and Jiang 2020 [13] ^1^ (*p* < 0.01)	KSE1 0.741		AVE 0.596					
	KSE2 0.795							
	KSE3 0.782							
	KSE4 0.769							
	KSE5 0.787							
	KSE6 0.755							
Present study 2024 ^2^ (*p* < 0.001)	KSE1 0.88		AVE 0.691					
	KSE2 0.83							
	KSE3 0.86							
	KSE4 0.87							
	KSE5 0.71							
	KSE6 0.82							

*Note.* RMSEA = root mean square error of approximation; CI = confidence interval; NFI = Bentler–Bonett Normed Fit Index; CFI = comparative fit index; IFI = Bollen’s Incremental Fit Index; TLI = Tucker–Lewis index; ^1^ first structure model includes openness and KS effects. ^2^ Data derived in the course of the present study.

**Table 2:** Comparison of the validity and reliability of strong ties and dense ties.

Strong Ties and Dense Ties (CFA)	χ^2^	χ^2^/df	RMSEA	SRMR	CFI	GFI	NFI	IFI	AVE	Cronbach Alpha
Models										
Song et al. 2019 [12]	χ^2^ = 1547.56 df = 209	χ^2^/df = 7.40	RMSEA = 0.161	SRMR = 0.11	CFI = 0.82	GFI = 0.64	NFI = 0.79	IFI = 0.82	Strong Ties = 0.655 Dense Ties = 0.459	Strong Ties = 0.878 Dense Ties = 0.765
Present study 2024 ^2^	χ^2^ = 37.796 df = 17 *p* = 0.003	χ^2^/df = 2.22	RMSEA = 0.112 CI-90 [0.064; 0.161] *p* = 0.021	SRMR = 0.033	CFI = 0.959	GFI = 0.979	NFI = 0.929	IFI = 0.96	Strong Ties = 0.681 Dense Ties = 0.542	Strong Ties = 0.903 Dense Ties = 0.834
Model comparison										
Comparison of strong ties	χ^2^-diff = 1510 df=-diff = 192 The *p*-value result is significant at *p* < 0.01.			deltaRMSEA = 0.049				deltaCFI = −0.139		

*Note*. RMSEA = root mean square error of approximation; CI = confidence interval; SRMR = standardized root mean square residual; CFI = comparative fit index; GFI = goodness-of-fit index; NFI = Bentler–Bonett Normed Fit Index; IFI = Bollen’s Incremental Fit Index; AVE = average variance extracted. ^1^ Bootstrapping 10.000 samples and maximum likelihood. ^2^ Data derived in the course of the present study.

**Table 3:** Comparison of acquisition of information, dissemination of information and use of information validity and reliability.

Models/Indices ESEM	χ^2^	χ^2^/df	RMSEA	SRMR	CFI	GFI	α	AVE
Model description and comparison								
Cheng and Shiu 2019 [3] ^1^		χ^2^/df = 2.01	RMSEA = 0.05		CFI = 0.91	GFI = 93		
Present study 2024 ^2^	χ^2^ = 47,453 df = 33 *p* = 0.049	χ^2^/df = 1.44	RMSEA = 0.067 CI-90 [0.003; 0.107] *p* = 0.243	SRMR = 0.0028	CFI = 0.974			
Comparison	Not calculable due to lack of data.			deltaRMSEA = −0.017		deltaCFI = 0.064		
Cheng and Shiu 2019 [3]	Acquisition of information						0.92	0.58
	Dissemination of information							
	Use of information							
Present study 2024 ^2^	Acquisition of information						0.81	0.48
	Dissemination of information						0.87	0.56
	Use of information						0.90	0.66

*Note*. RMSEA = root mean square error of approximation; CI = confidence interval; SRMR = standardized root mean square residual; CFI = comparative fit index; GFI = goodness-of-fit index; TLI = Tucker–Lewis index; AIC = Akaike information criterion. ^1^ In [3], ESEM analyses were calculated for model comparisons. ^2^ Data derived in the course of the present study.

## Data Availability

The raw data supporting the conclusions of this article will be made available by the authors on request. Further inquiries can be directed to the corresponding author.
